# *Trichoderma longibrachiatum* and *Aspergillus fischeri* Infection as a Cause of Skin Graft Failure in a Patient with Critical Burns after Liver Transplantation

**DOI:** 10.3390/jof7060487

**Published:** 2021-06-18

**Authors:** Břetislav Lipový, Filip Raška, Iva Kocmanová, Markéta Hanslianová, Martin Hladík, Jakub Holoubek, Matěj Bezdíček, Ctirad Macháček

**Affiliations:** 1 Department of Burns and Plastic Surgery, Institution Shared with University Hospital Brno, Faculty of Medicine, Masaryk University, 625 00 Brno, Czech Republic; bretalipovy@gmail.com (B.L.); raskafilip123@gmail.com (F.R.); holoubekjakub@yahoo.com (J.H.); 2 CEITEC—Central European Institute of Technology, Brno University of Technology, 612 00 Brno, Czech Republic; 3 Department of Clinical Microbiology, University Hospital Brno, 625 00 Brno, Czech Republic; kocmanova.iva@fnbrno.cz; 4 Department of Clinical Microbiology, Vyškov Hospital, 628 01 Vyškov, Czech Republic; hanslianova.marketa@nemvy.cz; 5 Centre of Molecular Biology and Gene Therapy, Department of Internal Medicine—Hematology and Oncology, Institution Shared with University Hospital Brno, Faculty of Medicine, Masaryk University, 625 00 Brno, Czech Republic; bezdicek.matej@fnbrno.cz; 6 Department of Pathology, Institution Shared with University Hospital Brno, Faculty of Medicine, Masaryk University, 625 00 Brno, Czech Republic; machacek.ctirad@fnbrno.cz

**Keywords:** dermal substitute, infection, *Trichoderma*, *Aspergillus*, critical burns, liver transplantation, immunosuppression

## Abstract

Infectious complications are responsible for the majority of mortalities and morbidities of patients with critical burns. Although bacteria are the predominant etiological agents in such patients, yeasts and fungi have become relatively common causes of infections over the last decade. Here, we report a case of a young man with critical burns on 88% TBSA (total body surface area) arising as a part of polytrauma. The patient’s history of orthotopic liver transplantation associated with the patient’s need to use combined immunosuppressant therapy was an additional complication. Due to deep burns in the forearm region, we have (after a suitable wound bed preparation) applied a new bi-layered dermal substitute. The patient, however, developed a combined fungal infection in the region of this dermal substitute caused by *Trichoderma longibrachiatum* and *Aspergillus fischeri* (the first case ever reported). The infection caused the loss of the split-thickness skin grafts (STSGs); we had to perform repeated hydrosurgical and mechanical debridement and a systemic antifungal treatment prior to re-application of the STSGs. The subsequent skin transplant was successful.

## 1. Introduction

For a long time, the treatment of patients with critical thermal trauma was almost solely oriented on survival. As a result, several key aspects were prioritized: fluid resuscitation, diagnosis and treatment of inhalation injury, organ support, infection control, and precision wound care, i.e., typically urgent necrectomy and application of several types of wound dressings, wound bed preparation, and subsequent permanent closure of the skin defect [1,2,3,4].

The dramatic improvement of the quality of acute care for critically burned patients led to the increase in the numbers of surviving patients with long-term consequences. For this reason, the emphasis is nowadays not placed just on the survival of such patients but also on the quality of their life after the trauma [5,6]. One of the principal aspects leading to the improvement of quality of life after a burn injury is the application of dermal substitutes [7,8]. The use of dermal substitutes followed by the application of STSGs (split-thickness skin grafts) helps preserve a very important functional property of the skin—viscoelasticity. Dermal substitutes maintain two functions of the cutaneous dermal layer—namely, the control of pain and scarring (wound healing and maturation). Generally, the application of dermal substitutes can be described as an effort to make the process of reparation closer to the process of regeneration.

Nevelia^®^ (Symatese Aesthetic, Chaponost, France) is a relatively new bi-layered dermal substitute. It consists of a three-dimensional porous matrix of Type I bovine collagen covered by a silicone layer reinforced with polyester, which represents pseudo-epidermis [9]. The pore size in the collagen matrix is optimized to approx. 100 µm, which supports fibroblast migration and neovascularization. The optimal time of collagen resorption is given by its balanced cross-linking rate (resorption takes 2–3 weeks from material implantation) [10]. Nevelia^®^ can be used not only for the treatment of burns (deep dermal, full-thickness burns) but also in reconstructive plastic surgery, traumatology (skin and soft tissue injuries of non-thermal aetiology), skin tumours, chronic wounds, and others [11].

This paper reports the first case of a combined fungal infection in the region of a bi-layered dermal substitute (Nevelia^®^) in a patient with critical burns and chronic immunosuppressant medication due to the orthotopic liver transplantation.

## 2. Case Report and Results

We present a case of a 20-year-old man who suffered a critical burn injury and polytrauma as a result of a car accident and subsequent fire. He was intubated on site, put on mechanical ventilation and transferred by air to the Department of Burns and Plastic Surgery of the University Hospital Brno.

An important aspect of the case is the patient’s medical history of liver transplantation two years ago due to primary biliary cirrhosis. The patient’s long-term medication includes calcineurin inhibitor (tacrolimus), mycophenolate mofetil and corticosteroids. After consulting the patient’s hepatologist, the immunosuppressant therapy was reduced to the necessary minimum (tacrolimus with regular checks to maintain plasma concentration within the range of 4–6 µg/L and hydrocortisone 100 mg/D). For the entire hospitalization period, the immunological condition of the patient was frequently monitored, focusing on the T-cell line and on the rate of CD14+ expression on the monocytes. CD3+ T-cell values were normal during hospitalization (0.70–2.10 × 10^9^/L); the CD4+/CD8+ ratio oscillated between 0.25 and 0.82. The CD14+ values on monocytes were in all measurements above 90%, a level signifying full immunocompetency.

Initially, a full-body spiral CT was performed according to the standard polytrauma algorithm, revealing multiple fractures of vertebrae and the sacral bone, contusion-induced changes in the right lung, infiltration in the vicinity of the spleen (subcapsular haematoma), and a shock bowel. Following consultation with traumatologists, it was decided that no urgent surgical treatment would be done and the fractures would be left to heal conservatively.

The initial examination was performed in the operating theatre in general anaesthesia. A central venous catheter and arterial line were inserted and the burns were treated. The total extent of the burns was 88% TBSA (total body surface area), where 80% were full-thickness burns (FTBs). In view of the location of the FTBs on the ventral side of the neck and adjacent areas, and of the expected long-term mechanical ventilation, an urgent surgical tracheostomy was performed. As a part of the complex diagnosis, bronchoscopy was performed and revealed Grade 1–2 inhalation injury.

After the primary treatment, the patient was hospitalized at the ICU of our department. The intensive fluid resuscitation continued, using continuously administered balanced crystalloid solutions combined with boluses of iso-oncotic human albumin (5% HA). Catecholamine support was needed due to circulatory instability. Active and passive tetanus immunization was also performed on admission. After the successful management of the initial burn shock, surgical treatment followed, including necrectomy, skin allotransplantation, wound bed preparation and wound closure using STSGs and, in some areas, application of dermal substitutes.

Surgical necrectomy of Grade III burns (80% TBSA) was gradually performed over the next few days; the defects were immediately provisionally covered with skin allotransplants (skin grafts from cadaverous donors). These allografts were used to minimize fluid losses through areas after necrectomy and to limit the possible microbial load. Once the condition of skin capable of spontaneous healing improved sufficiently to allow harvesting STSGs, allografts were removed and the wound bed was prepared and cleaned from avital structures. Subsequently, the wounds were covered with STSGs.

**Figure 1:**
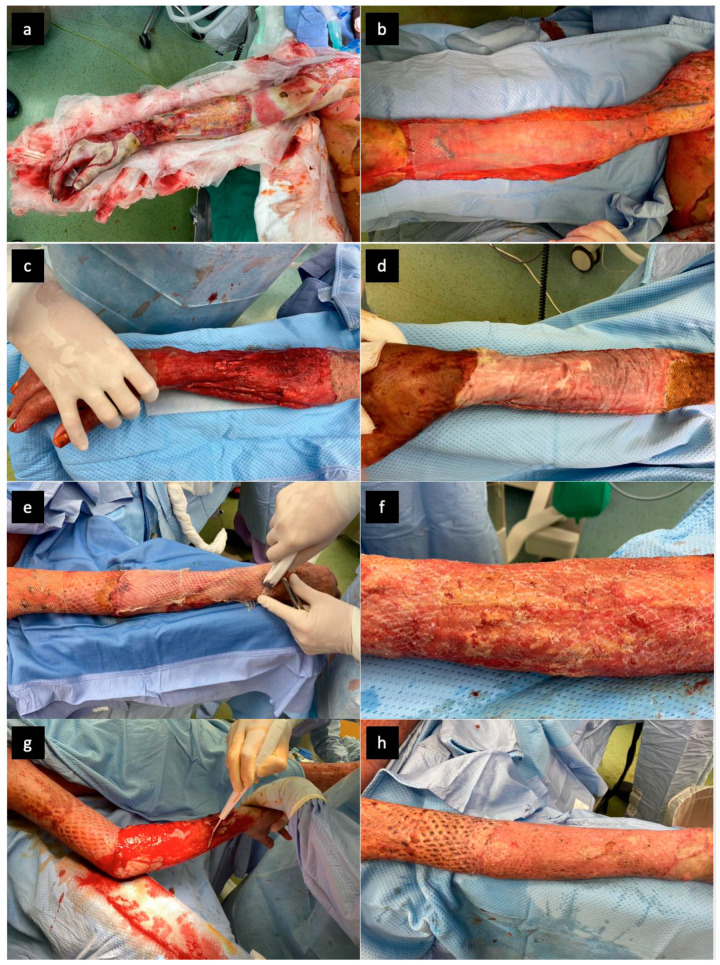
(**a**) Burn areas in the region of the upper right extremity on Day 2 after the injury, after surgical necrectomy; (**b**) following necrectomy, skin allograft was applied on the wound; (**c**) after wound bed preparation, a bi-layered dermal substitute (Nevelia^®^, Symatese Aesthetic, Chaponost, France) was applied; (**d**) the dermal substitute was fastened by clips and neovascularization gradually progressed; (**e**) then, autologous STSGs were applied; (**f**) loss of STSGs due to fungal infection; (**g**) hydrosurgical debridement prior to the next attempt for skin transplantation; (**h**) and healed STSG.

**Figure 2:**
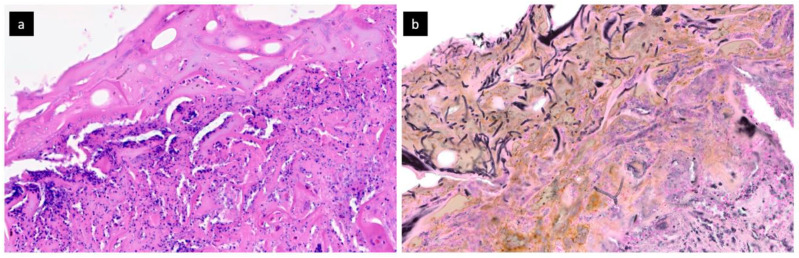
Histological staining: (**a**) Hematoxylin and eosin—superficial area of the skin graft with a layer of fibrin which contains mycotic hyphae, and granulation tissue with sufficient neovascularization is present in the lower right corner; (**b**) Grocott—mycotic hyphae highlighted by special staining are present in the superficial viable area without signs of invasive growth (superficial infection).

Due to the depth of the bilateral burns on the forearms, where important groups of muscles were exposed and partially destroyed, it was necessary to adopt a different approach to preserve the mobility of the fine forearm muscles below the reconstructed skin. Hence, a bi-layered dermal substitute (Nevelia^®^) was used in this location to minimize the functional damage to the upper extremities. After meticulous wound-bed preparation, the dermal substitute was applied to both forearms. Subsequent dressing changes showed the dermal substitute to firmly adhere to the wound bed, and no pathological secretion was detected on either of the upper extremities. After 3 weeks (time necessary for the revascularization of the dermal substitute), the upper silicon layer was removed and thin STSGs expanded at a 1:3 ratio were transplanted. The skin grafts on the left forearm gradually healed well but on the right forearm, they were largely lost due to infection (Figure 1) despite the dermal substitute maintaining the vital appearance and adhering well to the wound bed. Biopsy revealed sufficient neovascularization and presence of filamentous fungi in the region of the vital dermal substitute, without an obvious tendency to invade deeper soft tissues (Figure 2). Over the whole period of Nevelia^®^ application and graft loss, no other pathogens were detected in the problematic area. The debridement of lost transplants and remaining avital parts was gradually performed. Throughout the dermal substitute application, no bacterial co-infection was observed in the region of the right forearm. Only in the subsequent period of debridement, we isolated *Pseudomonas aeruginosa* in this region in amounts corresponding to bacterial colonization. Three weeks later, following negative bacterial and fungal culture results and repeated biopsy, re-transplantation of the STSG was performed. The grafts healed without any complications and with only minimum defects.

Regular microbiological surveillance with a targeted antimicrobial treatment was performed throughout the entire hospitalization period. Imprints from the burn wounds were regularly taken during dressing changes and subsequently semiquantitatively evaluated.

At the beginning of the hospitalization, these imprints were dominated by gram-positive cocci of the resident cutaneous microflora (*Staphylococcus epidermidis* and other coagulase-negative staphylococci, *Staphylococcus aureus* or beta-hemolytic streptococci). In the later stages, gram-negative rods (*Pseudomonas aeruginosa*, *Escherichia coli*, *Klebsiella oxytoca*, *Enterobacter cloacae*, *Acinectobacter baumannii*) dominated the microflora. Appropriate measures were always taken and the antimicrobial therapy adjusted.

As soon as in the first months of hospitalization, filamentous fungi were cultured from the patient’s burn wounds, predominantly on the lower extremities; namely, these included *Aspergillus fischeri* (closely related to a major human fungal pathogen, *Aspergillus fumigatus*) and *Trichoderma longibrachiatum*. As the amounts gave testimony rather about the colonization of the residual necrotic areas, we opted for local treatment only, i.e., debridement and application of antiseptics. After careful removal of all necroses, these fungi were no longer detected.

For mycological examinations, Sabouraud’s agar (Conda, Madrid, Spain) and incubation at 25, 30 and 37 °C were used. Identification of the strains was performed using panfungal PCR targeting the rDNA region with subsequent sequenation of the obtained PCR products following the protocol reported by Ferrer et al. [12]. The acquired sequences are stored in the GenBank^®^ database under the accession numbers MW647715 for *A. fischeri* and MW647703 for *T. longibrachiatum*.

**Figure 3:**
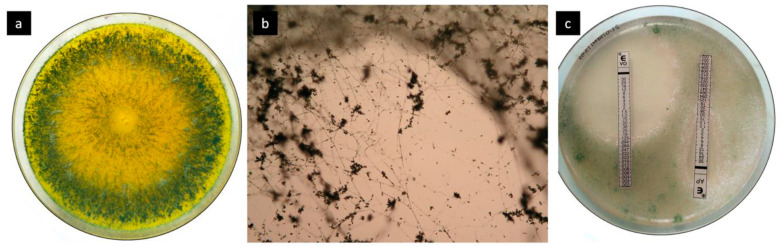
(**a**) Petri dish colonized by *T. longibrachiatum*, (**b**) microscopical view of *T. longibrachiatum*, (**c**) Etest of sensitivity to voriconazole (VO) and amphotericin (AP).

Sensitivity to antimycotic drugs was tested for both fungal species (amphotericin, voriconazole, posaconazole and isavuconazole) using the ETEST method (with RPMI agar 1640 containing 3-(N-morpholino)propanesulfonic acid and 2% glucose, read after incubation at 34–36 °C for 24 and 48 h). Achieved MIC (minimum inhibition concentrations) for *A. fischeri* were low (0.5, 0.25, 0.25, and 0.5 mg/L, respectively), with higher amounts recorded for *T. longibrachiatum* (0.5, 0.25, 2 and 2 mg/L) (Figure 3). Despite repeated capture in the skin defects, the filamentous fungi were not detected in other compartments. In weekly intervals, a panfungal antigen (1,3-beta-D-glucan, Fungitell™, ACCI, East Falmouth, MA, USA) and an aspergillus antigen (galactomannan, Platelia™Aspergillus Ag, BioRad, Marnes-la-Coquette, France) were analyzed in the serum, always with a negative result. Although the sensitivity of both these methods is lower in non-neutropenic patients, we still performed these tests to capture the potential mycotic infection. In view of the apparent non-invasiveness of the infection and a satisfactory clinical condition, fluconazole prophylactic treatment (immunocompromised patient) continued in the long term. After the grafts on the right forearm were lost, the antimycotic treatment was changed to voriconazole. This antimycotic screen gradually eradicated the *T. longibrachiatum* from the defects on the affected extremity and the acceptance of the STSG to the dermal substitute.

## 3. Discussion

Burn patients represent a high-risk group from the perspective of the development of infectious complications, including infections caused by opportunistic pathogens [13]. The everyday exposure to multiple pathogens makes infectious complications the predominant cause of mortality in these patients [14,15].

Infectious complications can manifest basically at any part of the body; nevertheless, the most common presentation is the burn-wound infection [16,17]. Burn-wound infections are most commonly caused by bacteria (70%), followed by yeasts and moulds (20–25%), and viruses (5–10%) [18].

In general, 20–44% of infections in burn patients are caused by fungi, depending on the geographical localization and the type of burn center [19,20,21].

The real incidence of fungal infections can, however, be higher; this hypothesis is based on the fact that many such infections are not properly identified and there is no clear clinical symptomatology of fungal wound infection (FWI) or colonization (FWC). If yeasts and/or moulds are detected in the region of the wound, it is necessary to strictly distinguish between FWC and FWI. FWC is defined as the identification of fungal elements in the burn necrosis not penetrating deeper into the deeper viable tissue. FWI, on the other hand, is defined as a fungal invasion into the viable tissue [22]. To distinguish between these two, it is necessary to perform a biopsy for histopathological examination; although it is an invasive procedure, it is essential for differentiating between these two conditions. Histopathological examination also provides the basic identification of the principal fungal morphology (aspergillus-like, mucor-like, or yeast-like morphology) [23].

Although the role of *Candida albicans* in the aetiology of infectious complications of critically ill patients gradually decreases, it remains the most common fungal infection in burn patients. A significant increase in the occurrence of filamentous fungi infections is, nevertheless, observed in the last decades [24].

Katz et al. describe cases of non-candidal fungal wound infection in acute burn patients [25]. According to their conclusions, this type of infectious complication is extremely rare (0.04%—12 cases out of 3340 patients). The most common among these were the *Aspergillus* spp. and *Fusarium* spp. Similar results were reported by Capoor et al. who also reported *Aspergillus* spp. and *Fusarium* spp. (2.8% and 1.4%, respectively) to be the most common causes of burn wound infections with filamentous fungi [26]. Rosanova et al. report a relatively high occurrence of *Fusarium* spp. infections in children with burn injuries, isolating these filamentous fungi in 15 out of 84 patients. In one of these patients, fusaria propagated into the bone and in another one, fungemia was reported [27].

Non-Aspergillus filamentous fungal (or mould) infections are increasingly reported, and despite that Fusaria and Zygomycota are the most common of these, they are not the only representatives causing such infections. The basic classification of non-Aspergillus filamentous fungi contains three groups: Mucormycetes, Phaeohyphomycetes, and Hyelohyphomycetes [28]. *Trichoderma* spp. belong to the hyelohyphomycetes group. These widespread filamentous fungi cause human infections only rarely [29]. Until recently, representatives of *Trichoderma* spp. were considered non-pathogenic for humans; however, *T. longibrachiatum* and *T. citrinoviride* are becoming emerging pathogens (in particular) in immunocompromised individuals [30,31]. Contaminated water and its aerosols are considered the most common source of contamination [32].

Infectious complications are characterized by the presence of fine septate hyphae (hyalohyphomycosis) in tissue sections. During differential diagnosis, they are often difficult to distinguish from invasive aspergillosis [33].

The most common site of isolation of *T. longibrachiatum* is the respiratory tract (up to 40%), followed by the skin [34]. In immunocompromised hosts, a wide range of infectious complications were described, including complicated skin and soft tissue infections (cSSTIs) or pneumonia, rarely causing also other infections such as sinusitis, peritonitis, endocarditis, brain abscess, keratitis, mediastinitis, liver infection, stomatitis, infection of cardiac implantable electronic device, or disseminated infections [35,36,37,38,39,40]. So far, no infectious complications caused by *T. longibrachiatum* have been reported and recorded in burn patients.

The principal risk factors for the development of fungal burn-wound infection include the age, extent of the burn area, inhalation injury, presence of full-thickness burns, use of broad-spectrum antibiotics, prolonged hospitalization period, diabetes, total parenteral nutrition (TPN), fungal wound colonization, immunosuppressant therapy, and application of dermal substitutes [41,42].

Many studies focused on infectious complications associated with application of dermal substitutes, reporting infectious rates of 6 to 88%, depending on the type of wound, surgical procedure (acute or reconstruction surgery), condition of the patient, and type of dermal substitute [43,44,45,46,47,48].

The cause of the fungal infection in this patient is unclear. Of course, we cannot exclude the possibility that the fungal infection was transferred to the region of interest together with the skin graft. Nevertheless, the filamentous fungi were isolated only in the burn wounds with necroses; wherever the burns healed, no fungal infection was detected anymore. Moreover, the graft was harvested from the upper half of the body from a site where there never was any necrosis and microbiological surveillance never detected any fungal infection or colonization in that region; furthermore, in sites where skin grafts were applied directly on the prepared wound bed without the use of dermal substitute, no fungal infection occurred. Therefore, we believe that this mode of transmission is (although possible) unlikely.

In our opinion, the most likely explanation was an infection of the Nevelia^®^ dermal substitute during manipulation (dressing changes, etc.) during the period when it was not yet properly vascularized and, therefore, the immune system of the patient (even an immunosuppressed one) that would otherwise be able to deal with the infection was unable to suppress it. However, we took swabs from the Nevelia^®^ dermal substitute just before skin graft application, and this swab did not reveal any fungal colonization. Therefore, if the infection was present just before the skin graft application, it would have to be present only in negligible amounts in the superficial layer of Nevelia^®^ and only developed after the application of the skin graft.

Patients with thermal trauma represent a very vulnerable group from the perspective of the development of infectious complications as a specific form of immunosuppression, corresponding to the severity of the thermal trauma, occurs in these patients. Immunosuppressant therapy present even before the injury further potentiates the problem of trauma-induced immunosuppression and leads to a further dramatic increase in the development of infectious complications caused by opportunistic pathogens.

Immunosuppressants used in our patient (tacrolimus and corticosteroids) may cause wound healing impairment. They influence inflammatory mediators involved in wound healing such as IL-2, IL-4, interferon-γ, and tumor necrosis factor-α [49]. Tacrolimus also decreases systemic nitric oxide synthesis, which is important for the tissue healing process, and suppresses T-cell activation through the inhibition of calcineurin and the calcineurin-dependent transcription factor. Corticosteroids are well known to delay wound healing by interfering with inflammatory, proliferative, and remodeling processes [50]. For these reasons, we also monitored, besides the plasma concentration of tacrolimus, the hepatic transaminase levels and immunocompetence with a special focus on the T-cell line and CD14+ on monocytes. The role of immunosuppression in the development of infectious complications in this patient is difficult to evaluate as multiple factors have probably contributed towards it. However, reason dictates that in such patients, it is absolutely essential to individualize the microbiological surveillance in such patients and to immediately react to its results.

If yeasts and/or filamentous fungal infection are detected, it is also important to combine the pathogen proof by cultivation with testing of the presence of fungal antigens at regular intervals to be able to assess the dynamic development over time (as single time-point inflammatory markers might not always provide sufficient evidence). Similarly, the clinical symptomatology of the local or systemic infection can be, due to the immunosuppression, significantly compromised.

In these patients, therefore, targeted antimicrobial treatment combined with regular debridement and appropriate wound bed preparation are essential for successful wound closure using STSG.

## Data Availability

The data presented in this study are available on request from the corresponding author.

## References

[1] Kao Y., Loh E., Hsu C., Lin H., Huang C., Chou Y., Lien C., Tam K. (2018). Fluid Resuscitation in Patients With Severe Burns: A Meta-analysis of Randomized Controlled Trials. Acad. Emerg. Med..

[2] Gacto-Sanchez P. (2017). Surgical treatment and management of the severely burn patient: Review and update. Med. Intensiv..

[3] Vigani A., Culler C.A. (2017). Systemic and Local Management of Burn Wounds. Vet. Clin. N. Am. Small Anim. Pract..

[4] Rowley-Conwy G. (2013). Management of burns in intensive and acute care. Nurs. Stand..

[5] Spronk I., Legemate C., Oen I., Van Loey N., Polinder S., Van Baar M. (2018). Health related quality of life in adults after burn injuries: A systematic review. PLoS ONE.

[6] Kool M.B., Geenen R., Egberts M.R., Wanders H., Van Loey N.E. (2017). Patients’ perspectives on quality of life after burn. Burns.

[7] Shahrokhi S., Arno A., Jeschke M.G. (2014). The use of dermal substitutes in burn surgery: Acute phase. Wound Repair Regen..

[8] van der Veen V.C., van der Wal M.B., van Leeuwen M.C., Ulrich M.M., Middelkoop E. (2010). Biological background of dermal substitutes. Burns.

[9] Uccioli L., Meloni M., Izzo V., Giurato L. (2020). Use of Nevelia Dermal-Epidermal Regenerative Template in the Management of Ischemic Diabetic Foot Postsurgical Wounds. Int. J. Low. Extrem. Wounds.

[10] De Angelis B., Orlandi F., D’Autilio M.F.L.M., Scioli M.G., Orlandi A., Cervelli V., Gentile P. (2018). Long-term follow-up comparison of two different bi-layer dermal substitutes in tissue regeneration: Clinical outcomes and histological findings. Int. Wound J..

[11] Yiğitbaş H. (2019). Our experience with dermal substitute Nevelia^®^ in the treatment of severely burned patients. Turk. J. Trauma Emerg. Surg..

[12] Ferrer C., Colom F., Fraseés S., Mulet E., Abad J.L., Alioó J.L. (2001). Detection and Identification of Fungal Pathogens by PCR and by ITS2 and 5.8S Ribosomal DNA Typing in Ocular Infections. J. Clin. Microbiol..

[13] Lipový B., Brychta P., Řihová H., Hanslianová M., Loskotová A., Jarkovský J., Kaloudová Y., Suchánek I. (2016). Prevalence of infectious complications in burn patients requiring intensive care: Data from a pan-European study. Epidemiol. Mikrobiol. Imunol..

[14] Ceniceros A., Pértega S., Galeiras R., Mourelo M., Lopez E., Broullon J., Sousa L., Freire D., Pértega-Díaz S. (2015). Predicting mortality in burn patients with bacteraemia. Infection.

[15] Egozi D., Hussein K., Filson S., Mashiach T., Ullmann Y., Raz-Pasteur A. (2013). Bloodstream infection as a predictor for mortality in severe burn patients: An 11-year study. Epidemiol. Infect..

[16] Church D., Elsayed S., Reid O., Winston B., Lindsay R. (2006). Burn Wound Infections. Clin. Microbiol. Rev..

[17] Lachiewicz A.M., Hauck C.G., Weber D.J., Cairns A.B., Van Duin D. (2017). Bacterial Infections After Burn Injuries: Impact of Multidrug Resistance. Clin. Infect. Dis..

[18] Horvath E.E., Murray C.K., Vaughan G.M., Chung K.K., Hospenthal D.R., Wade C.E., Holcomb J.B., Wolf S., Mason A.D., Cancio L.C. (2007). Fungal Wound Infection (Not Colonization) Is Independently Associated with Mortality in Burn Patients. Ann. Surg..

[19] Becker W.K., Cioffi W.G., McManus A.T., Kim S.H., McManus W.F., Mason A.D., Pruitt B.A. (1991). Fungal Burn Wound Infection. Arch. Surg..

[20] Mousa A.H. (1999). Fungal infection of burn wounds in patients with open and occlusive treatment methods. East. Mediterr. Health J..

[21] Murray C.K., Loo F.L., Hospenthal D.R., Cancio L.C., Jones J.A., Kim S.H., Holcomb J.B., Wade C.E., Wolf S. (2008). Incidence of systemic fungal infection and related mortality following severe burns. Burns.

[22] Howard P., Cancio L., McManus A., Goodwin C., Kim S., Pruitt B. (1999). What’s new in burn-associated infections?. Curr. Surg..

[23] Schofield C.M., Murray C.K., Horvath E.E., Cancio L.C., Kim S.H., Wolf S., Hospenthal D.R. (2007). Correlation of culture with histopathology in fungal burn wound colonization and infection. Burns.

[24] Schaal J., Leclerc T., Soler C., Donat N., Cirrode A., Jault P., Bargues L. (2015). Epidemiology of filamentous fungal infections in burned patients: A French retrospective study. Burns.

[25] Katz T., Wasiak J., Cleland H., Padiglione A. (2014). Incidence of non-candidal fungal infections in severe burn injury: An Australian perspective. Burns.

[26] Capoor M.R., Gupta S., Sarabahi S., Mishra A., Tiwari V.K., Aggarwal P. (2011). Epidemiological and clinico-mycological profile of fungal wound infection from largest burn centre in Asia. Mycoses.

[27] Rosanova M.T., Brizuela M., Villasboas M., Guarracino F., Alvarez V., Santos P., Finquelievich J. (2016). Fusarium spp infections in a pediatric burn unit: Nine years of experience. Braz. J. Infect. Dis..

[28] Douglas A.P., Chen S.C.-A., Slavin M.A. (2016). Emerging infections caused by non- Aspergillus filamentous fungi. Clin. Microbiol. Infect..

[29] Chouaki T., Lavarde V., Lachaud L., Raccurt C.P., Hennequin C. (2002). Invasive Infections Due toTrichodermaSpecies: Report of 2 Cases, Findings of In Vitro Susceptibility Testing, and Review of the Literature. Clin. Infect. Dis..

[30] Kredics L., Antal Z., Dóczi I., Manczinger L., Kevei F., Nagy E. (2003). Clinical importance of the genus Trichoderma. Acta Microbiol. Immunol. Hung..

[31] Trabelsi S., Hariga D., Khaled S. (2010). First case of Trichoderma longibrachiatum infection in a renal transplant recipient in Tunisia and review of the literature. Tunis Med..

[32] Chen S.C.-A., Blyth C.C., Sorrell T.C., Slavin M. (2011). Pneumonia and Lung Infections due to Emerging and Unusual Fungal Pathogens. Semin. Respir. Crit. Care Med..

[33] Walsh T., Groll A., Hiemenz J., Fleming R., Roilides E., Anaissie E. (2004). Infections due to emerging and uncommon medically important fungal pathogens. Clin. Microbiol. Infect..

[34] Sandoval-Denis M., Sutton D.A., Cano-Lira J.F., Gené J., Fothergill A.W., Wiederhold N.P., Guarro J. (2014). Phylogeny of the Clinically Relevant Species of the Emerging Fungus Trichoderma and Their Antifungal Susceptibilities. J. Clin. Microbiol..

[35] Furukawa H., Kusne S., Sutton D.A., Manez R., Carrau R., Nichols K.A.L., Skedros D., Todo S., Rinaldi M.G., Nichols L. (1998). Acute invasive sinusitis due to Trichoderma longibrachiatum in a liver and small bowel transplant recipient. Clin. Infect. Dis..

[36] Kerr C.M., Perfect J.R., Craven P.C., Jorgensen J.H., Drutz D.J., Shelburne J.D., Gallis H.A., Gutman R.A. (1983). Fungal Peritonitis in Patients on Continuous Ambulatory Peritoneal Dialysis. Ann. Intern. Med..

[37] Piens M.A., Celard M., de Monbrison F., Grando J., Vandenesch F., Mottolese C., Picot S. (2004). Trichoderma infection of cerebro-spinal fluid shunt device in a non immunocompromised patient. J. Mycol. Med..

[38] Ranque S., Garcia-Hermoso D., Michel-Nguyen A., Dumon H. (2008). Isolation of Trichoderma atroviride from a liver transplant. J. Mycol. Méd..

[39] Seguin P., Degeilh B., Grulois I., Gacouin A., Maugendre S., Dufour T., Dupont B., Camus C. (1995). Successful treatment of a brain abscess due toTrichoderma longibrachiatum after surgical resection. Eur. J. Clin. Microbiol. Infect. Dis..

[40] Tascini C., Cardinali G., Barletta V., Di Paolo A., Leonildi A., Zucchelli G., Corte L., Colabella C., Roscini L., Consorte A. (2015). First Case of Trichoderma longibrachiatum CIED (Cardiac Implantable Electronic Device)-Associated Endocarditis in a Non-immunocompromised Host: Biofilm Removal and Diagnostic Problems in the Light of the Current Literature. Mycopathologia.

[41] Struck M., Gille J. (2013). Fungal infections in burns: A comprehensive review. Ann. Burn. Fire Disasters.

[42] Kyriopoulos E., Kyriakopoulos A., Karonidis A., Gravvanis A., Gamatsi I., Tsironis C., Tsoutsos D. (2015). Burn injuries and soft tissue traumas complicated by mucormycosis infection: A report of six cases and review of the literature. Ann. Burn. Fire Disasters.

[43] Gonzalez S.R., Wolter K.G., Yuen J.C. (2020). Infectious Complications Associated with the Use of Integra: A Systematic Review of the Literature. Plast. Reconstr. Surg. Glob. Open.

[44] Peck M.D., Kessler M., Meyer A.A., Morris P.A.B. (2002). A Trial of the Effectiveness of Artificial Dermis in the Treatment of Patients with Burns Greater Than 45% Total Body Surface Area. J. Trauma Acute Care Surg..

[45] Munster A.M., Smith-Meek M., Shalom A. (2001). Acellular allograft dermal matrix: Immediate or delayed epidermal coverage?. Burns.

[46] Branski L.K., Herndon D.N., Pereira C., Mlcak R.P., Celis M.M., Lee J.O., Sanford A.P., Norbury W.B., Zhang X.-J., Jeschke M.G. (2007). Longitudinal assessment of Integra in primary burn management: A randomized pediatric clinical trial. Crit. Care Med..

[47] Bloemen M.C.T., Van Der Wal M.B.A., Verhaegen P.D.H.M., Nieuwenhuis M., Van Baar M.E., Van Zuijlen P.P.M., Middelkoop E. (2012). Clinical effectiveness of dermal substitution in burns by topical negative pressure: A multicenter randomized controlled trial. Wound Repair Regen..

[48] Lagus H., Sarlomo-Rikala M., Böhling T., Vuola J. (2013). Prospective study on burns treated with Integra^®^, a cellulose sponge and split thickness skin graft: Comparative clinical and histological study—Randomized controlled trial. Burns.

[49] Bootun R. (2012). Effects of immunosuppressive therapy on wound healing. Int. Wound J..

[50] Zhang H., Qu W., Nazzal M., Ortiz J. (2020). Burn patients with history of kidney transplant experience increased incidence of wound infection. Burns.

